# Supplementary material for: A Simple and Effective Visual Fluorescent Sensing Paper-Based Chip for the Ultrasensitive Detection of Mercury Ions in Environmental Water
Source: Sensors (Basel). 2023 Mar 14;23(6):3094. doi: 10.3390/s23063094 (PMC10058424; doi:10.3390/s23063094)
Supplement: Supplementary file 1 [file sensors-23-03094-s001.zip › sensors-2191547-supplementary.pdf]

Supplementary Information

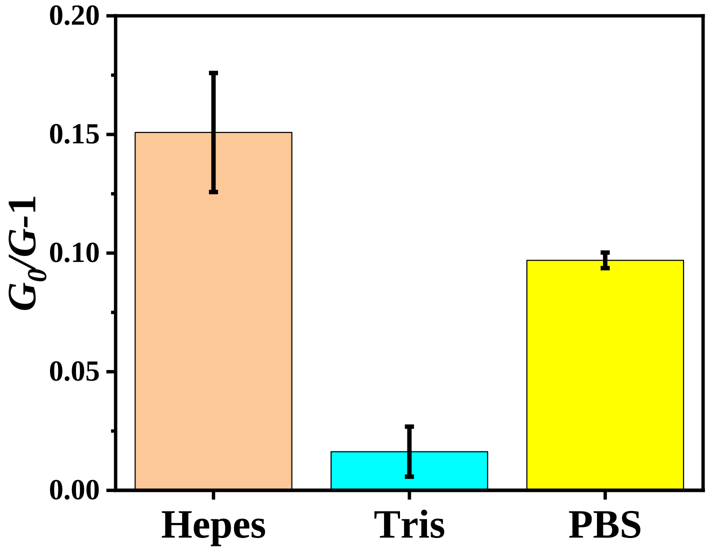

Figure S1. Influence of buffer system on quantum dot quenching effect. The effect of fluorescence quenching can be fitted by the following equation: (G_0/G)-1, G_0 is the gray value of the graph before the drop of Hg^{2+} is added, G is the gray value of the graph after the drop of 50 μg/L Hg^{2+}. All buffer system concentrations were 0.01M, pH were 7.5. The experimental conditions were room temperature.

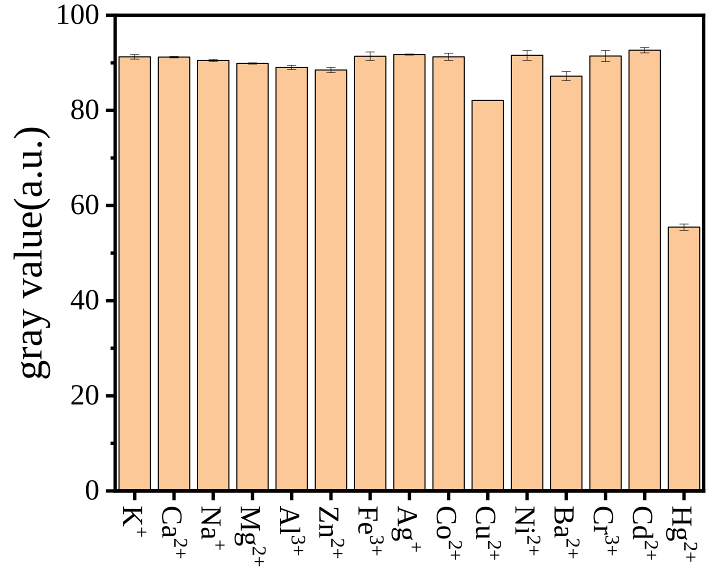

Figure S2. Selectivity for different interfering substances. The concentration of all interferents were 50 μg/L. The experimental conditions were at room temperature and pH were 7.5.

Table S1. Performance comparison with other reported methods for paper-based visual detection of Hg^{2+}

Colorimetric system	Linear rang	LOD	Sample matrices	References
**CdSe/ZnS QDs-based catalytic TMB oxidation**	0.02-20.05 mg/L	18.05 μg/L	Cosmetic cream and tap water	[11]
**MAA capped CdSAg QDs**	24.87-160.47 mg/L	24.87 mg/L	Cosmetic acid solution	[27]
**MOF/ CdTe QDs**	10~5000 mg/L	0.22 mg/L	red wine	[28]
**Rhodamine-Vinyl ether probe**	10-200 mg/L	1mg/L	Tap water	[14]
**N-CQDs**	0.2-102.51mg/L	0.16 mg/L	Lake wate r	[12]
**AgNPs**	40-1200 μg/L	10 μg/L	Industrial wastewater and river water	[10]
**Paper-based QDs-SiO_2**	5-100 μg/L	2.83 μg/L	Seawater and environmental water	This work
